# Modelling bark thickness for Scots pine (Pinus sylvestris L.) and common oak (Quercus robur L.) with recurrent neural networks

**DOI:** 10.1371/journal.pone.0276798

**Published:** 2022-11-16

**Authors:** Dominika Cywicka, Agnieszka Jakóbik, Jarosław Socha, Daryna Pasichnyk, Adrian Widlak

**Affiliations:** 1 Faculty of Computer Science and Telecommunications, Cracow University of Technology, Cracow, Poland; 2 Department of Forest Resources Management, Faculty of Forestry, the University of Agriculture in Krakow, Cracow, Poland; Hanyang University, KOREA, REPUBLIC OF

## Abstract

Variation of the bark depends on tree age, origin, geographic location, or site conditions like temperature and water availability. Most of these variables are characterized by very high variability but above of all are also affected by climate changes. This requires the construction of improved bark thickness models that take this complexity into account. We propose a new approach based on time series. We used a recurrent neural network (ANN) to build the bark thickness model and compare it with stem taper curves adjusted to predict double bark thickness. The data includes 750 felled trees from common oak and 144 Scots pine—trees representing dominant forest-forming tree species in Europe. The trees were selected across stands varied in terms of age and site conditions. Based on the data, we built recurrent ANN and calculated bark thickness along the stem. We tested different network structures with one- and two-time window delay and three learning algorithms—Bayesian Regularization, Levenberg-Marquardt, and Scaled Conjugate Gradient. The evaluation criteria of the models were: coefficient of determination, root mean square error, mean absolute error as well as graphical analysis of observed and estimated values. The results show that recurrent ANN is a universal approach that offers the most precise estimation of bark thickness at a particular stem height. The ANN recursive model had an advantage in estimating trees that were atypical for height, as well as upper and lower parts on the stem.

## 1 Introduction

The bark is an outer covering of the stems and roots of trees. Its role is to protect the tree against mechanical damage, high temperature, and excessive transpiration. It is produced by phellogen and is mainly made up of dead cells. On young trees, the bark is smooth—it thickens with age, begins to crack, and falls off. These changes are species-specific and depend on external factors. From an economical point of view, bark can be important as a fuel and a source of valuable biomaterials [1]. However, in forestry, the key issue is to calculate bark thickness due to timber volume estimation in forest inventories or log trade [2]. Modeling bark is mainly based on the identification of factors influencing the variability of its thickness. These include tree age, origin, geographic location, and altitude or site conditions. Currently, the mechanical role of the bark is essential in weather conditions, which are characterized by violent thunderstorms, and strong winds. Changes in average temperatures may also result in changes in the thickness of the bark of trees, as this tissue actively participates in transpiration. The phenomenon of bark thickness reaction to temperature was observed in tree species exposed to high temperatures and fire [3]. Changes taking place in the environment in recent years are intense, which induces the need to build new models. Within a single tree, modeling the thickness of the bark along the stem is related to its elliptical growth. Directional variation in bark thickness was observed in pine. It is thicker to the north and thinner to the south [4]. The surface of the bark has a complex pattern—it is not smooth but rough and irregular. This makes it difficult to estimate the thickness along the trunk. Regression solutions are most often used to describe the double bark thickness (DBT). Another approach is to adapt the taper models so that bark thickness is the dependent variable. [5]. However, such nonlinear functions tend to smooth the shape. This results in an inaccurate description—usually in the lower or top part of the tree [6]. Moreover, the course of the taper function is different for the stem than for the bark itself. The course of the variability of the function describing the thickness of the bark along the tree trunk seems to have visible fluctuations, which may be random, and not only the trend related to the tapering of the stem with height. For individual trees, the dynamics of this function can be complicated—not only non-linear but also non-periodic. However, the individual observations of the series—the thickness of the bark at different tree heights—are interrelated. Such dynamic processes can be analyzed with a time series approach. Artificial neural networks (ANN) can be used to implement such relationships. Neural networks replace time-series decomposition with a learning process. With ANN can effectively describe relationships that are difficult to model with analytical methods. ANN is resistant to disturbances and adapts better to local conditions [7, 8]. Hornik, Stinchcombe and White [9] proved that multilayer perceptron networks with one hidden layer could theoretically be considered universal approximators. Neural networks have found application in forest resource management [10] for estimating tree diameter, height, volume, growth, and mortality of trees and others [11–14]. So far, no attempt has been made to use a recursive neural network to estimate bark thickness. In the presented research, tree species of high importance were selected for analysis. Pine (Pinus sylvestris L.) is one of the key species of European forests. Its range covers the boreal region of Northern and Eastern Europe to the mountains of the Mediterranean in Southeastern Europe. In Poland, it currently covers approximately 58 percent of the forest area [15]. Oak (Quercus L.) is the main species of the second-largest site type—European mixed forest. It is predicted that the range of the thermophilic species, resistant to drought or strong winds, may increase [16, 17]. Consequently, its economic importance will also be more significant. The thickness of the bark is a feature that can be thought of as an evolutionary adaptation to high temperatures. An increase in average annual temperatures [18] entails the need to adopt new approaches dedicated to modeling bark thickness. Developing such new models may be particularly important under changing climate conditions. Additionally, those equations are essential to quantify tree biomass, which is closely related to the supply of ecosystem services. The results of this study are clues for forest management decisions aimed at balancing timber and non-timber objectives.

This study aimed to (i) estimate bark thickness variations within pine and oak stands using a recurrent neural network, and (ii) compare it with adopted stem taper models. As a reference for ANN model, we used two polynomial regression equation models developed by Cao et al. [19], and improved by Li et al. [6].

Contributions to the proposed work include building a new recursive model of bark thickness for the most important tree species in Europe. Different bark thickness models based on neural networks will be estimated and compared.

## 2 Material and methods

### 2.1 Study area

**Fig 1:**
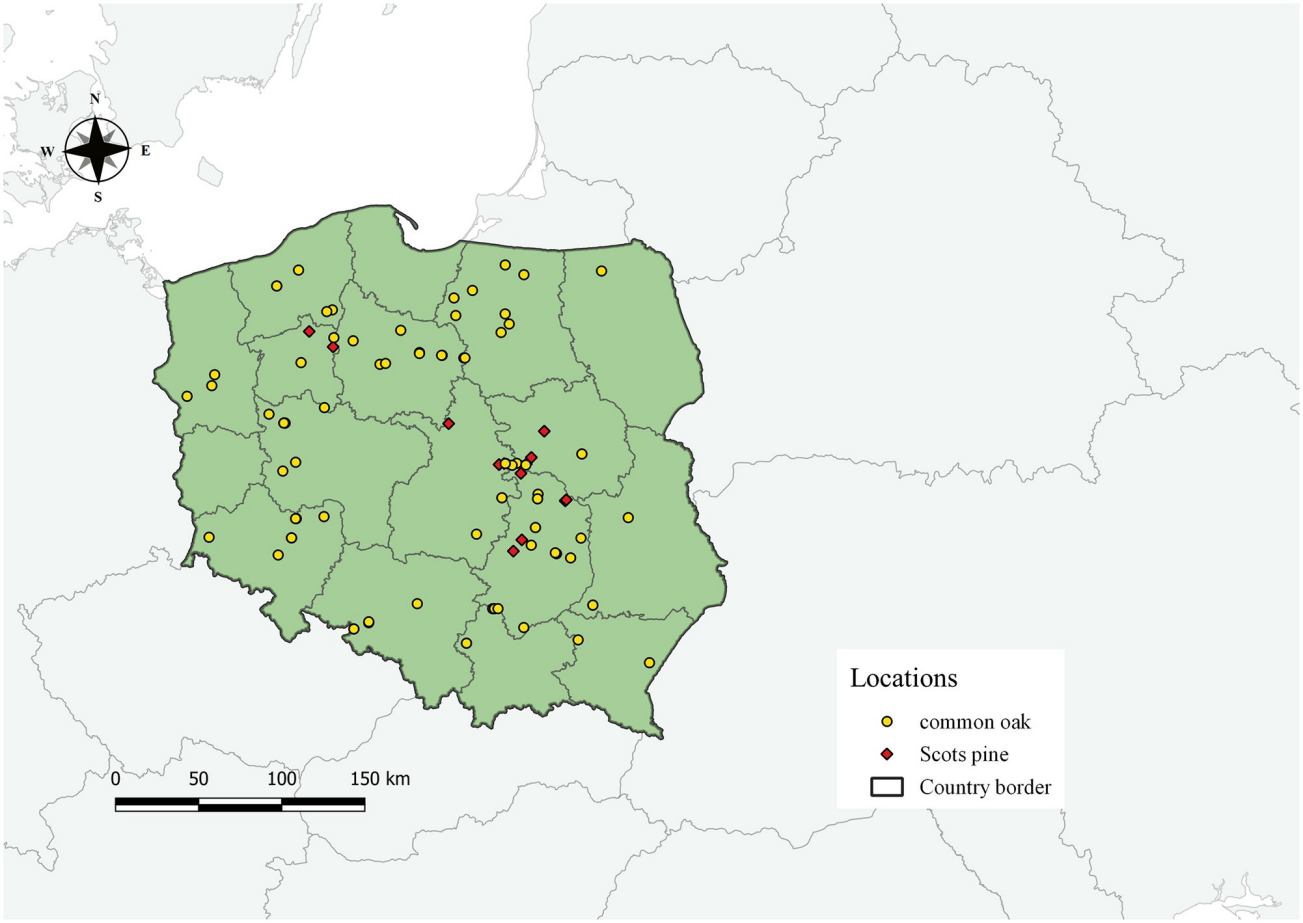
Selected sample plots distribution for scots pine and common oak in Poland.

Data for this study were collected from selected trees growing on 99 sample plots distributed throughout Poland in Central Europe (Fig 1). Poland has a moderate climate with both maritime and continental elements, with annual temperature and rainfall averages of 6–8°C and 700 mm, respectively (https://klimat.imgw.pl).

### 2.2 Data obtaining

In each plot, at least 20 trees of the main species with a diameter at breast height (DBH) larger than 7 cm were selected. Further, on the basis of their DBH, nine most representative sample trees were selected for the sectional measurements of thickness and bark ranging from the lowest to the highest DBH. Altogether, a total of 750 common oak (Quercus robur L.) and 144 Scots pine (Pinus sylvestris L.) trees were selected for the above purpose. After felling, diameter measurements (cm) were taken directly with calipers at different tree heights: cutting height—0.0, then 0.5; 1.3 and 2.0 m from the ground level. The consecutive measurements were taken every 2 m up to the tree top. Bark thickness was measured with a bark thickness gauge with an accuracy of 1 mm in two perpendicular directions of stem thickness measurements, as follows: at the base of the stem (at the point of truncation—i.e., at the bottom face) and at the centers of the sections where stem diameter in the bark was measured. The diameter without bark (cm) was obtained by subtracting the double bark thickness from the diameter outside bark.

### 2.3 Artificial Neural Networks based prediction method

Making predictions using Artificial Neural Networks is done using a data-driven approach. This analysis method consumes only the collected data; no additional or previous knowledge of modeled phenomenon is used. The first stage of the proposed approach was to train an appropriately selected Artificial Neural Network based on the measurements that were collected in the field: that is *DBT*, *H*, *H*_*b*_(*k*), see Table 1. This process enables ANN to adjust its inner parameters (weights) to model the given tree’s bark thickness according to the desired, known values that the teacher presents. The black box model is built for calculating desired target bark three based on given tree measurements. Any additional information like tree type, known mathematical models, or tree location is not included. After the training phase, Neural Network was given the data that was not presented during learning, that is a new set of trees. For those trees, a prediction was made. It was based on the parameters that were calculated for the trees that were presented so far.

**Table 1:** Characteristics of the data. *k*_1_(*cm*)—bark thickness at height *H*_*b*_, *k*_2_(*cm*)—bark thickness at height *H*_*b*_, *DBT*(*cm*)—double bark thickness at height *H*_*b*_, *H*_*b*_(*m*)—height along the bole from the ground, *H*_*b*_(*m*)—height along the bole from the ground, *DOB*(*cm*)—stem diameter outside bark at height *H*_*b*_.

Variable (unit)	Range pine [min, max]	Range oak [min, max]
*k*_1_(*cm*)	0.05,6.50	0.01,5.50
*k*_2_(*cm*)	0.05,6.50	0.01,5.40
*DBT*(*cm*)	0.10,13.00	0.03,10.91
*H*(*m*)	18.30,32.70	13.90,35.90
*H*_*b*_(*m*)	0.00,32.00	0.00,36.00
*DOB*(*cm*)	0.60,75.55	0.25,129.25

#### 2.3.1 Adapting time series approach for the tree data set

**Fig 2:**
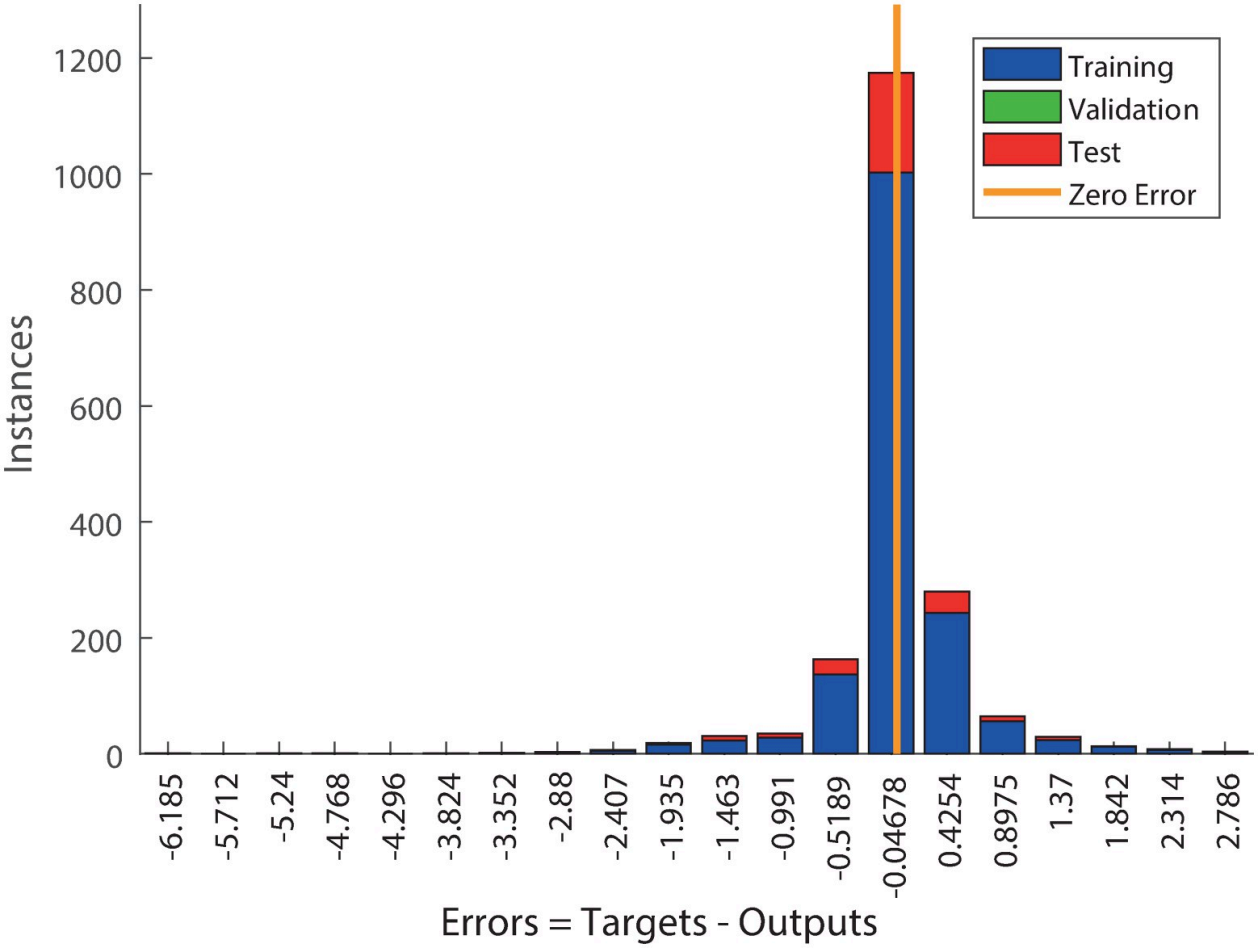
First 100 data vectors from the numerical experiment evaluated for pine. Black cycles indicate value from the point where the diameter was measured to the tip of the tree, blue cross indicates *avg*_*d*_ and the NARX ANN target is depicted as a red dashed line. Horizontal axis [1]—identifier of measurement.

The measurements of the *DOB* value for the particular three in relation to the modeled double bark thickness *k*_1_ + *k*_2_ sequentially were ordered tree by tree (see Fig 2). It can be observed that the measurements may be interpreted as the time series for the independent variable *k* ∈ *N* equal to the consecutive number of samples. This series is decreasing repetitively for each new tree. For the reason that *H* value for the particular tree is constant and *H*_*b*_ is a growing value, an additional variable (*DTT*) determined by both *H* and *H*_*b*_ was introduced as follows:

$$\begin{array}{c} DTT\left(k\right)=H-H_{b}\left(k\right) \end{array}$$ (1)

where *k* = 1, …, 1842 number of measurement. This relates to both the tree height and measurement point and its value is decreasing as the *DOB* value decreases and may be interpreted as the distance from the measured diameter at absolute height to the tip of the tree.

In time series modeling, nonlinear autoregressive exogenous models (NARX) are used when a series depends strongly on its own past values. Using a nonlinear autoregressive model allows introduction the very complex nature of such dependencies. NARX models also incorporate exogenous inputs. These externally determined variables influence the series. In addition, the model contains an “error” term, which relates to the fact that knowledge of other terms is not known precisely. Such a model can be stated as:

$$\begin{array}{c} y_{k}=F\left(y_{k-1},y_{k-2},\ldots ,y_{k-N},\ldots ,u_{k},u_{k-1},u_{k-2},\ldots ,u_{k-M},\ldots \right)+\varepsilon_{t} \end{array}$$ (2)

where in our case *y*(*k*) = *k*_1_ + *k*_2_(*k*) is the dependent variable of interest, and *u* is the externally variable, *ε* is the error term. The function F is nonlinear. In our approach F is assumed in the form of a neural network. The numbers of delays *N*, *M* define a time window for dependent and independent variables. The rest of the variables are assumed according to formulas presented in the next subsection.

#### 2.3.2 NARX ANN with time window delay

To model the estimated double bark thickness variation we used a nonlinear autoregressive exogenous Neural Network model (NARX ANN)with sigmoidal activation functions in the hidden layer and linear activation function for the output, [20].

Then, the following inputs and targets for NARX ANN were introduced, see Fig 2.

$$\begin{array}{c} ANN_{input}\left(k\right)=\left[avg_{d}\left(k\right),DDT\left(k\right)\right] \end{array}$$ (3)

$$\begin{array}{c} ANN_{target}\left(k\right)=\left[\left(k_{1}\left(k\right)+k_{2}\left(k\right)\right)\right] \end{array}$$ (4)

Such a design of the input data stream resulted in the same cyclic descending values as within the target data stream. Assuming

$$\begin{array}{c} u_{k}=ANN_{input}\left(k\right) \end{array}$$ (5)

it completes the definition of the model (2). In order to verify the quality of modeling the mean squared error (MSE) and R coefficient of variation were used for the learning and testing process separately:

$$\begin{array}{c} MSE=\sum_{k=1,\ldots ,K}{\left(ANN_{target}\left(k\right)-ANN_{output}\left(k\right)\right)}^{2}/K \end{array}$$ (6)

where K is the number of samples (K = 1564 for pine learning process, K = 276 for pine testing process, K = 10027 for oak learning process K = 1769 for oak testing process).

Nguyen-Widrow initialization method was used for the initialization of all ANN starting weights, [21]. As the preprocessing of the data linear Min-Max scaler was used to standardize all the data into [0, 1] intervals. Three different learning methods were examined, including Bayesian Regularization training (BR), Levenberg-Marquardt algorithm (LM), and Scaled Conjugate Gradient (SCG), [22]. After introductory testing the considered size of the time window delay for input and output, were either two past values included or a single past value included—it results in *N*, *M* ∈ {1, 2}. Repeated random sub-sampling validation was used for each ANN. 15% of randomly chosen data from the learning data set was used for model validation. Validation sets are used to stop training early if the network performance on the validation vectors fails to improve. Additionally, one round of cross-validation was used to compare the results across different ANNs with the same number of internal connections. All data from the testing set was used for cross-validation. Matlab R2017 solver was used to conduct any necessary numerical experiments [23].

Two layered NARX ANN were examined with a different number of neurons in the hidden layer, see Table 4. The set of collected data was divided into a training set, containing 70% of data, a testing set, and validation sets containing respectively 15% of all data. For the pine, it resulted in 1288 samples for training, 276 for validation, and 276 for testing. For oak 8258 samples were used for training and 1769 for validation and 1769 for testing. The data were scaled to [0, 1] interval in the prepossessing stage, and the results of ANN simulation were scaled back to the original data range. Both MSE error and correlation coefficient R were calculated for those three sets separately in order to exclude possible negative over-fitting effects. Each ANN network type, presented in Table 4, was trained 100 times with different initial weight sets. The dependence between initial weights and final training results after meeting the stopping criteria was observed insignificant. All the networks were trained with different starting weights. The different sets of starting weights were stated not to impact the obtained MSE errors significantly. The standard deviation of MSE value equaled less than 0.1%. Considering cross-validation between ANNs of the same structure, the standard deviation of MSE value for different cross-validation sets was observed less than 2%. Therefore we decided to present the final results for the network that was randomly chosen from the set of 100 previously trained ANNs.

#### 2.3.3 Comparison with bark taper models

In order to compare results from NARX ANN modeling, the following two statistically obtained functions were introduced—STAT_1_ and STAT_2_. The taper model selected in this study was first proposed by Cao and Pepper [19] to model diameter inside bark for three pine species and introduced by Li and Weiskittel [6] for modeling double bark thickness. It turned out that these equations have the best results of all the compared models. The equations differ by adding quotient term: diameter inside bark at breast height/ diameter outside bark at breast height. This activity was intended to increase the performance of the model by reducing bias. From a practical point of view, this quotient information is (diameter inside bark at breast height) rarely available in forest inventory databases [6]. The dependent variable of equations STAT_1_ and STAT_2_ was changed in this study to describe double bark thickness. In the original study, it performs diameter inside the bark.

Let the

$$\begin{array}{c} DBT=k_{1}+k_{2} \end{array}$$ (7)

be the Double Bark Thickens calculated based on field measurements and

$$\begin{array}{c} ANN=ANN_{output} \end{array}$$ (8)

be the value of Double Bark Thickens simulated by ANN.

$$\begin{array}{c} STAT_{\text{1}}= \end{array}$$ (9)

$$\begin{array}{c} avg_{d}*\left(b_{1}+b_{2}\frac{Hb(k)}{H}+b3\left({\frac{Hb(k)}{H}}^{2}+b_{4}*H\right)\right)+b_{5} \end{array}$$ (10)

where the coefficients are listed in Table 2.

**Table 2:** The coefficients of statistical models (9)-(10).

Three	*b*_1_	*b*_2_	*b*_3_	*b*_4_	*b*_5_
pine	0.155363	−0.344976	0.317674	−0.001186	−0.060022
oak	0.108146	0.054412	−0.003357	−0.001480	0.488477

$$\begin{array}{c} STAT_{\text{2}}= \end{array}$$ (11)

$$\begin{array}{c} avg_{d}*({\bar{b}}_{1}+{\bar{b}}_{2}\frac{Hb(k)}{H}+{\bar{b}}_{3}{\left(\frac{Hb(k)}{H}\right)}^{2}+{\bar{b}}_{4}*H+\ldots \end{array}$$ (12)

$$\begin{array}{c} \ldots +{\bar{b}}_{5}\left(\left(d_{1_{1}3}+d_{2_{1}3}\right)/2-\left(k_{1_{1}3}+k_{2_{1}3}\right)\right)/\left(\left(d_{1_{1}3}+d_{2_{1}3}\right)/2\right))+{\bar{b}}_{6} \end{array}$$ (13)

where the coefficients are listed in Table 3.

**Table 3:** The coefficients of statistical models (11)-(12).

Three	${\bar{b}}_{1}$	${\bar{b}}_{2}$	${\bar{b}}_{3}$	${\bar{b}}_{4}$	${\bar{b}}_{5}$	${\bar{b}}_{6}$
pine	0.525331	-0.342769	0.310562	-0.001421,	-0.407817	0.020500
oak	0.669494	0.054381	0.003500	-0.000408,	-0.644409	0.404019

Were calculated using Statistica solver [Statistica 13.1 software].

In order to compare those models, the following measures were used.

Root mean squared error:

$$\begin{array}{c} RMSE\left(cm\right)=\sqrt{\sum_{k=1,\ldots ,K}{\left(DBT\left(k\right)-DBT_{i}\left(k\right)\right)}^{2}/K} \end{array}$$ (14)

Summed error in the form of:

$$\begin{array}{c} ME\left(cm\right)=\sum_{k=1,\ldots ,K}\left(DBT\left(k\right)-DBT_{i}\left(k\right)\right)/K \end{array}$$ (15)

Model efficiency coefficient:

$$\begin{array}{c} EF\left(cm\right)=1-\frac{(\sum_{k=1,\ldots ,K}{\left(DBT\left(k\right)-DBT_{i}\left(k\right)\right)}^{2}}{(\sum_{k=1,\ldots ,K}{\left(DBT_{i}\left(k\right)-MEAN\left(DBT_{i}\right)\right)}^{2}} \end{array}$$ (16)

Mean error:

$$\begin{array}{c} MEAN\left(DBT\right)\left(cm\right)=\frac{\sum_{k=1,\ldots ,K}DBT_{i}\left(k\right)}{K} \end{array}$$ (17)

$$\begin{array}{c} MAE=\frac{\sum_{k=1,\ldots ,K}\left|\left(DBT\left(k\right)-DT_{i}\left(k\right)\right)\right|}{K} \end{array}$$ (18)

where *i* ∈ {*STAT*, *ANN*}.

**Fig 3:**
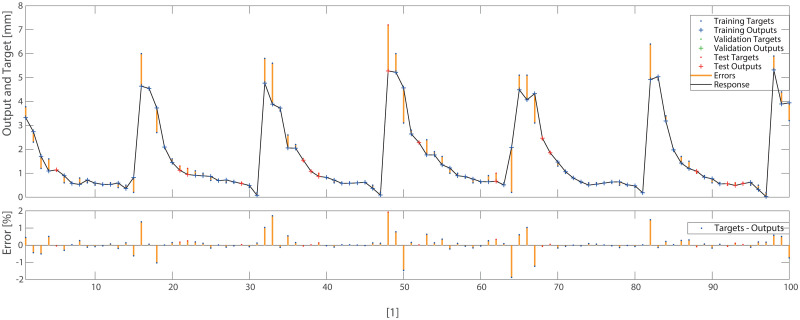
Histogram of ANN errors with 20 bins for 2(1)-20–1 networked trained with BR, evaluated for pine data.

**Fig 4:**
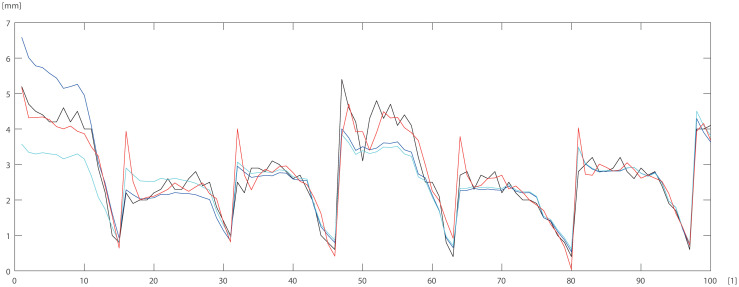
Results of simulation for 2(1)-20–1 networked trained with BR for pine data, where horizontal axis [1]—identifier of measurement.

The sample values obtained from the Neural Network model are depicted in Fig 4. The errors are presented on Fig 3.

## 3 Results

The best results for modelling the thickness of pine bark were obtained for NARX 2(1)-20–1 ANN trained by the Bayesian Regularization algorithm (Table 4). Smaller ANN resulted in a correlation coefficient R-value in the testing set lower than R = 9.0. By introducing additional time delay in the input and output layers, we received a lower MSE value for smaller ANNs trained by Bayesien Regularization algorithm. In this case, the counterproductive result for both: LM and SCG training methods, were received. Using a time delay of length 2 for bigger ANNs results in ANN over-fitting using BR. For ANN trained by LM results were similar to those using smaller ANN but the training lasts longer. SCG training was stated not very efficient for bigger NARX ANN with a time delay value equal 2.

**Table 4:** BR—Bayesian Regularization training, LM -Levenberg-Marquardt training algorithm, SCG—Scaled Conjugate Gradient, the number in the brackets indicates the size of the time window delay for input and output, (2) two past values included, or (1) single past value included, evaluated for pine data set, see Fig 2.

	*MSE*	*R*	*MSE*	*R*	*MSE*	*R*	
**ANN**	**2(1)-10–1 BR**		**2(1)-10–1 LM**		**2(1)-10–1 SCG**		%
*Training*	3.55e-1	9.36e-1	3.56e-1	9.361e-1	7.11e-1	8.72e-1	70
*Validation*	0e-0	0e-0	6.49e-1	8.96e-1	5.52e-1	8.99e-1	15
*Testing*	5.73e-1	8.87e-1	6.28e-1	8.80e-1	5.79e-1	8.83e-1.	15
**ANN**	**2(2)-10–1 BR**		**2(2)-10–1 LM**		**2(2)-10–1 SCG**		%
*Training*	2.33e-1	9.58e-1	4.33e-1	9.21e-1	1.31e-0	7.59e-0	70
*Validation*	0e-0	0e-0	7.83e-1	8.64e-1	9.16e-1	8.17e-1	15
*Testing*	5.69e-1	8.87e-1	6.88e-1	8.70e-1	1.22e-0	7.44e-1	15
**ANN**	**2(1)-20–1 BR**		**2(1)-20–1 LM**		**2(1)-20–1 SCG**		%
*Training*	3.40e-1	9.39e-1	5.03e-1	9.06e-1	7.13e-1	8.69e-1	70
*Validation*	0e-0	0e-0	3.6e-1	9.34e-1	6.68e-1	8.67e-1	15
*Testing*	4.91e-1	9.11e-1	7.41e-1	8.81e-1	6.17e-1	8.95e-1	15
**ANN**	**2(2)-20–1 BR**		**2(2)-20–1 LM**		**2(2)-20–1 SCG**		%
*Training*	2.05e-1	9.63e-1	4.50e-1	9.16e-1	1.22e-0	7.70	70
*Validation*	0e-0	0e-0	6.23e-1	9.04e-1	1.21e-0	7.85e-1	15
*Testing*	3.91e-0	6.85e-1	5.92e-1	8.98e-1	1.21e-0	7.26e-1	15

The aim of the first numerical experiment was to conclude if the training method had an influence in the results obtained (see first three rows of Tables 4 and 5). It resulted in the conclusion that the Bayesian Regularization training was the most effective for a given data sets for NARX 2(1)-10–1 ANNs. During the next experiment, the second delay into ANN output into input was introduced. We concluded that introducing a second delay was not much beneficial for obtaining the best results however supported ANN trained by LM and SCG algorithms to lower MSE errors. The most significant improvement was stated for SCG learning method. Forth experiment was conducted in order to detect if adding additional neurons in the hidden layer without adding a second delay unit can improve the results. We concluded that for BR and SCG algorithms this strategy enables lowering the value of MSE error. In the fourth experiment, we tested larger networks (20 hidden neurons) with two delay units, which gave better results for BR and LM training algorithms. Additionally in this case we obtained the best results among all tested cases for NARX 2(1)-20–1 ANN trained by the BR algorithm. Those results were similar for both pine and oak training sets. For a given model form, a combination of the BR algorithm with NARX 2(1)-20–1 generally produced a more reliable prediction of bark thickness profile (Fig 4).

**Table 5:** BR—Bayesian Regularization training, LM -Levenberg-Marquardt training algorithm, SCG—Scaled Conjugate Gradient, the number in the brackets indicates the size of the time window delay for input and output, (2) two past values included, or (1) single past value included, evaluated for oak.

	*MSE*	*R*	*MSE*	*R*	*MSE*	*R*	
**ANN**	**2(1)-10–1 BR**		**2(1)-10–1 LM**		**2(1)-10–1 SCG**		%
*Training*	4.05e-1	8.94e-1	4.22e-1	8.86e-1	8.34–1	7.62e-1	70
*Validation*	0e-0	0e-0	4.08e-1	8.96e-1	9.23e-1	7.45e-1	15
*Testing*	4.01e-1	8.82e-1	4.60e-1	8.73e-1	8.80e-1	7.34e-1	15
**ANN**	**2(2)-10–1 BR**		**2(2)-10–1 LM**		**2(2)-10–1 SCG**		%
*Training*	3.75e-1	8.99e-1	3.81e-1	8.97e-1	5.41e-0	8.52e-1	70
*Validation*	0e-0	0e-0	4.69e-1	8.78e-1	5.52e-1	8.53e-1	15
*Testing*	4.67e-1	8.79e-1	4.19e-1	8.90e-1	5.62e-0	8.494e-1	15
**ANN**	**2(1)-20–1 BR**		**2(1)-20–1 LM**		**2(1)-20–1 SCG**		%
*Training*	4.02e-1	8.93e-1	5.44e-1	8.54e-1	5.95e-1	8.36e-1	70
*Validation*	0e-0	0e-0	5.78e-1	8.40e-1	6.13e-1	8.42e-1	15
*Testing*	4.36e-1	8.79e-1	5.19e-1	8.55e-1	5.86e-1	8.33e-1	15
**ANN**	**2(2)-20–1 BR**		**2(2)-20–1 LM**		**2(2)-20–1 SCG**		%
*Training*	3.805e-1	8.99e-1	14e-1	8.90e-1	8.08e-0	7.74e-1	70
*Validation*	0e-0	0e-0	4.16e-1	8.93e-1	7.83e-0	7.78e-1	15
*Testing*	4.03e-0	8.93e-1	3.91e-1	8.89e-1	8.51e-0	7.44e-1	15

For the purpose of this research, three different types of NARX ANN were tested. They differed in both the number of hidden neurons and the number of delays for input and output. The time window delay was two past values included, or a single past value included. All types of ANN were trained based on three different learning methods: BR—Bayesian Regularization training, LM -Levenberg-Marquardt training algorithm, and SCG—Scaled Conjugate Gradient, see Tables 4 and 5. Bayesian regularization is a process that converts a nonlinear regression into a statistical problem. LM algorithm interpolates between the Gauss-Newton algorithm and the method of gradient descent. SCG proceeded in a direction that is conjugated with the directions of the previous steps.

Tables 6 and 7 demonstrate that the best results for both oak and pine regarding RMSE and MAE had the ANN model. The other two models were comparable and approximately two times worse than the neural one. STAT_2_ had the highest efficiency for both species, but there were no significant differences between the neural and STAT_1_ in this respect. The difference in EF between STAT_2_ and ANN was 0.05 (for pine) and 0.02 (for oak). The STAT_1_ for pine was worse than the neural model by 0.01, but for oak, the difference was considerable—0.25. Statistical models obtained the lowest value of ME error.

**Table 6:** Accuracy measurements of the artificial neural network model (ANN), the polynomial regression equations (STAT_1_ and STAT_2_) for the estimation of the double bark thickness for pine.

	ANN	STAT_1_	STAT_2_
**RMSE**	0.6440	1.2739	1.2984
**ME**	0.3681	0.0330	-0.0067
**EF**	0.8389	0.8249	0.8782
**MAE**	0.3335	0.4148	0.3652

**Table 7:** Accuracy measurements of the artificial neural network model (ANN), the polynomial regression equations (STAT_1_ and STAT_2_) for the estimation of the double bark thickness for oak.

	ANN	STAT_1_	STAT_2_
**RMSE**	0.6348	1.6874	1.6874
**ME**	0.0031	-0.0005	0.0008
**EF**	0.7591	0.5080	0.7752
**MAE**	0.4002	0.5988	0.4646

**Fig 5:**
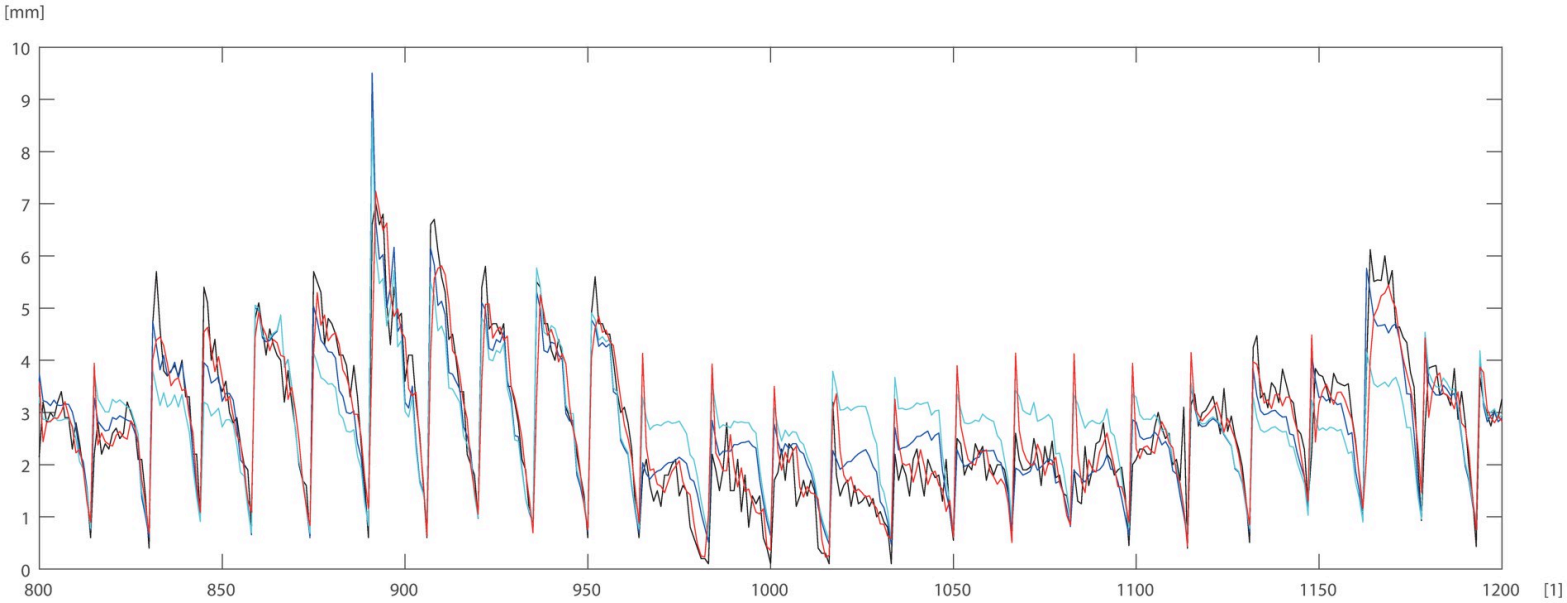
150 DBT data (black line), STAT_2_ (blue line), STAT_1_ (cyan line), ANN (red line) for pine. Horizontal axis [1]—identifier of measurement.

**Fig 6:**
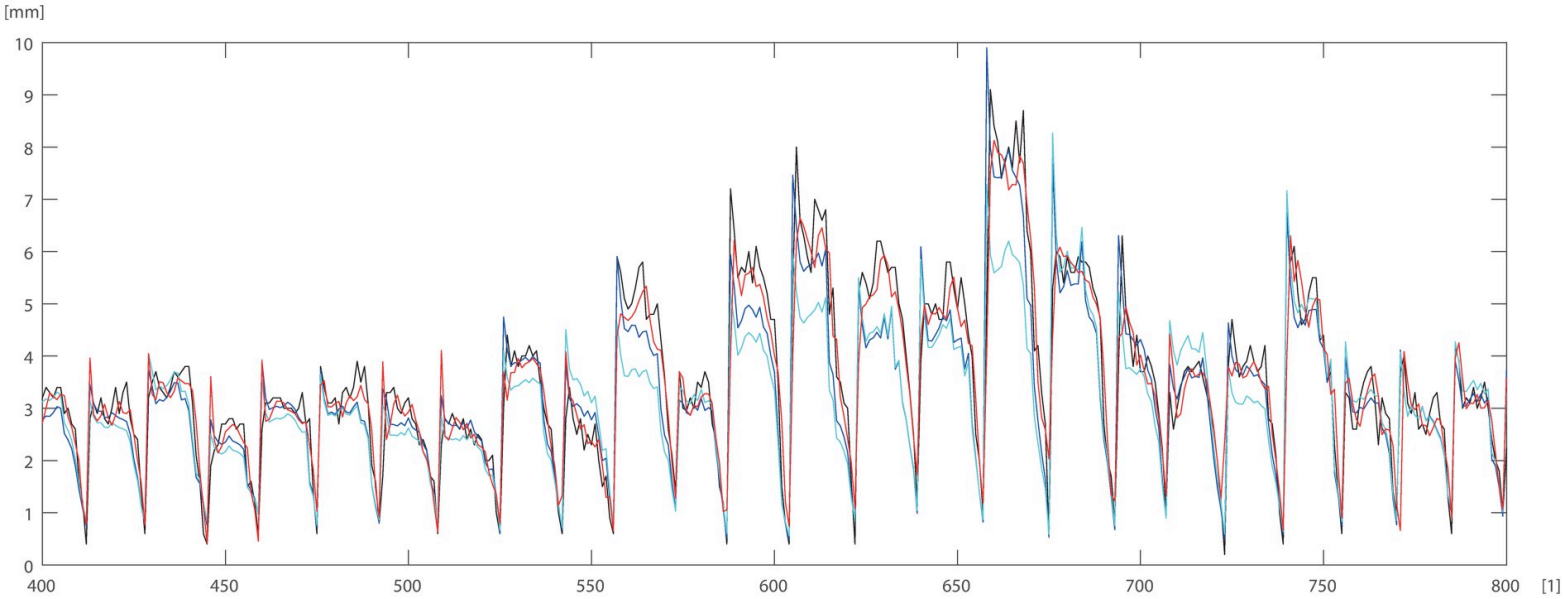
150 DBT data (black line), STAT_2_ (blue line), STAT_1_ (cyan line), ANN (red line) for pine. Horizontal axis [1]—identifier of measurement.

**Fig 7:**
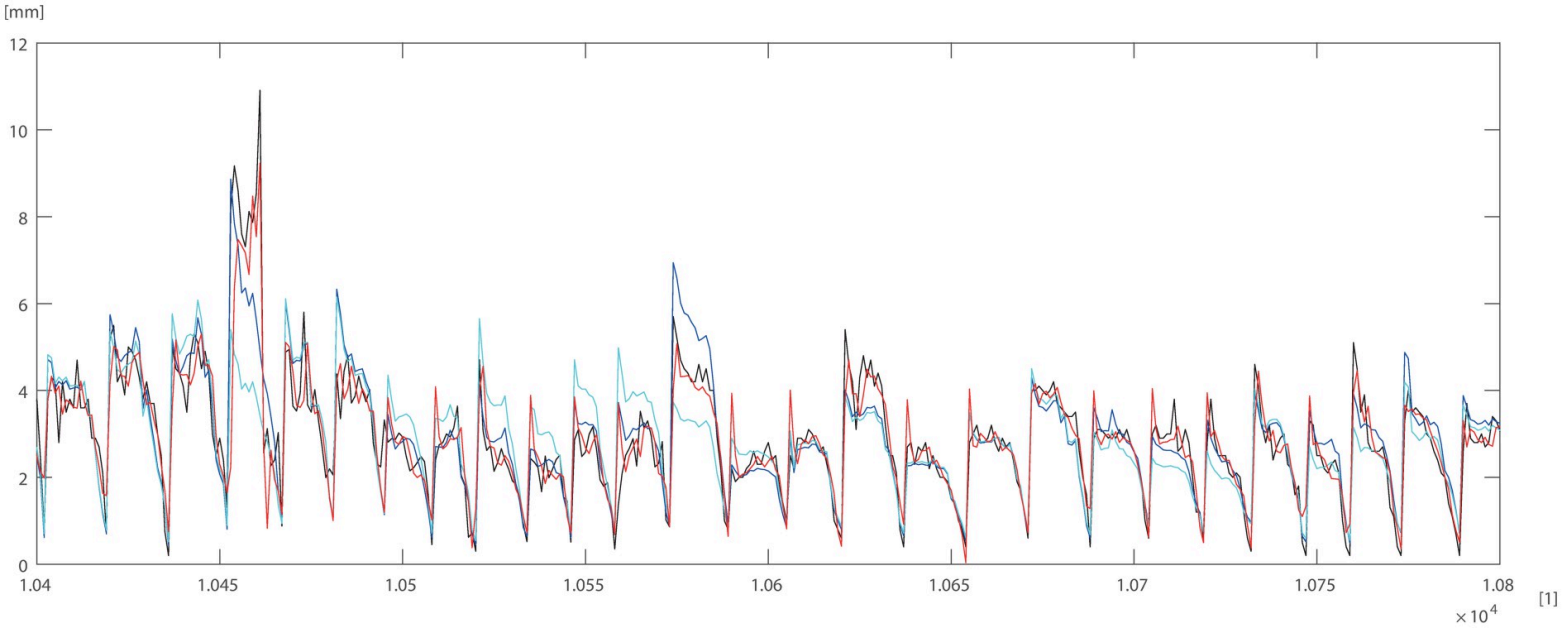
First 60 DBT data (black line), STAT_2_ (blue line), STAT_1_ (cyan line), ANN (red line) for pine. Horizontal axis [1]—identifier of measurement.

**Fig 8:**
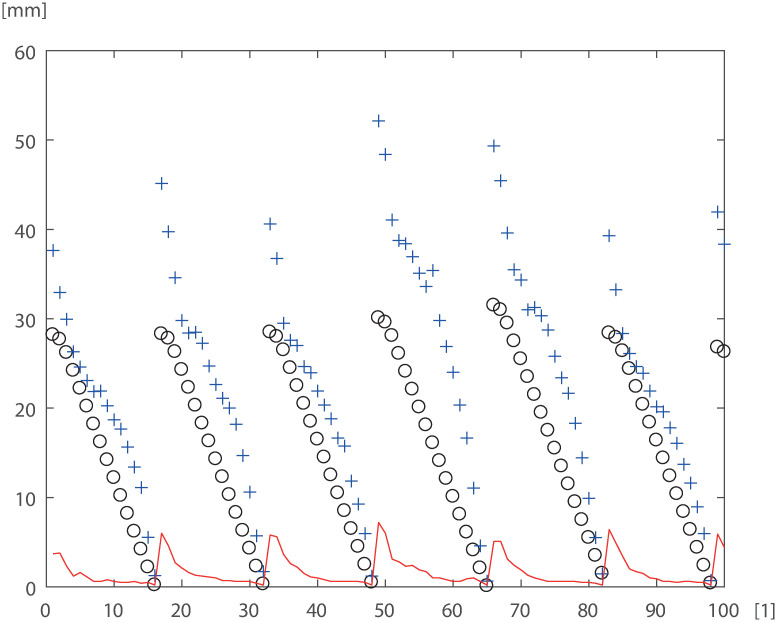
100 DBT data (black line), STAT_2_ (blue line), STAT_1_ (cyan line), ANN (red line) for oak. Horizontal axis [1]—identifier of measurement.

**Fig 9:**
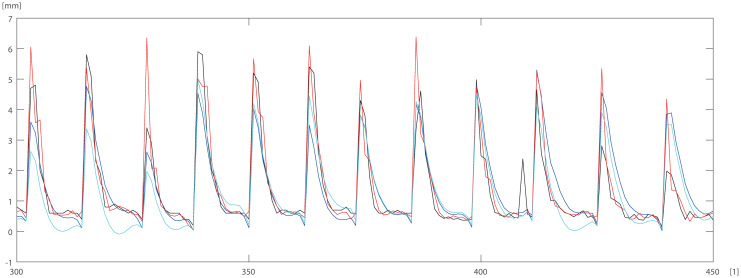
400 DBT data (black line), STAT_2_ (blue line), STAT_1_ (cyan line), ANN (red line) for oak. Horizontal axis [1]—identifier of measurement.

**Fig 10:**
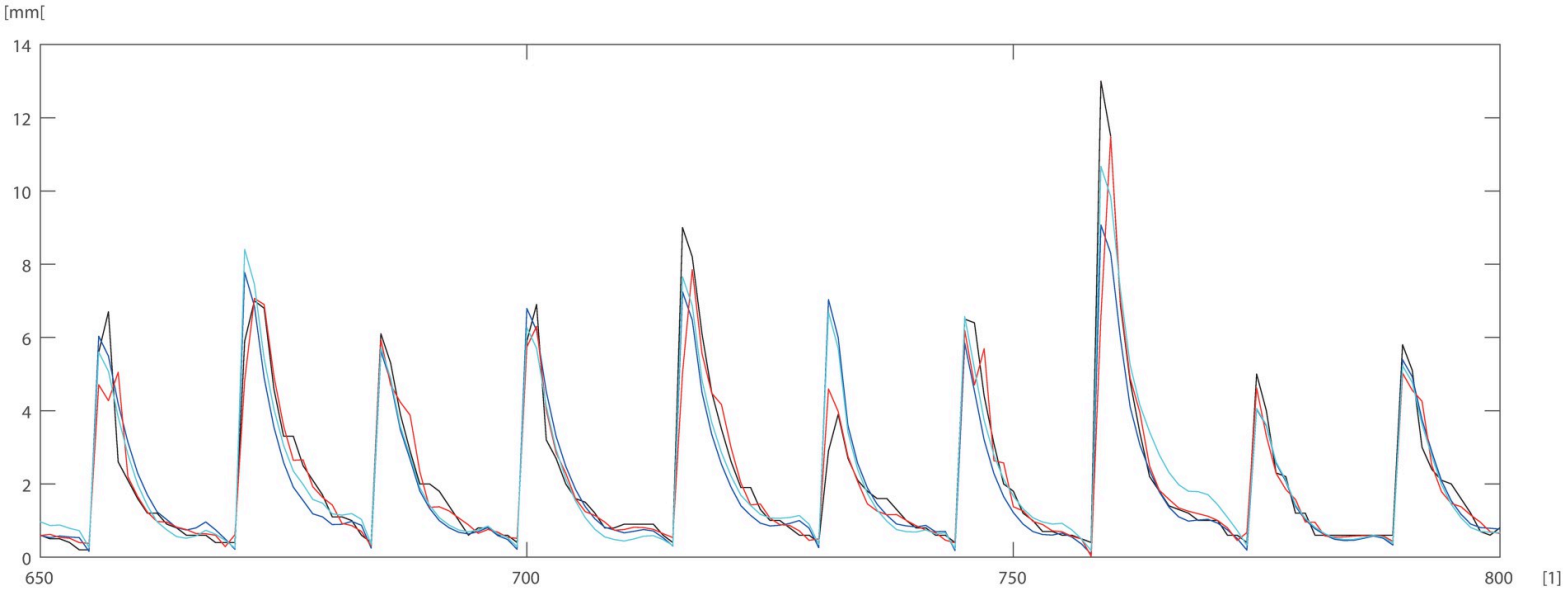
400 DBT data (black line), STAT_2_ (blue line), STAT_1_ (cyan line), ANN (red line) for oak. Horizontal axis [1]—identifier of measurement.

**Fig 11:**
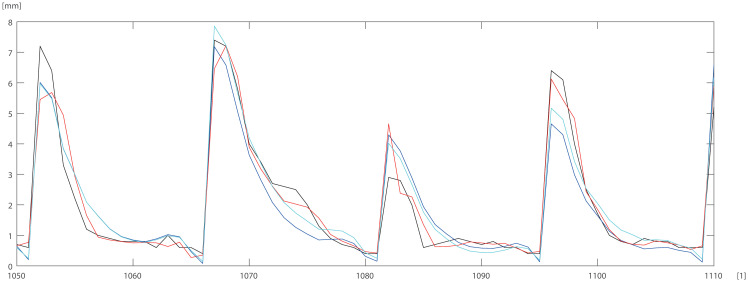
400 DBT data (black line), STAT_2_ (blue line), STAT_1_ (cyan line), ANN (red line) for oak. Horizontal axis [1]—identifier of measurement.

To check and compare the models, we plotted a sample of data for individual trees. The differences between DBT data obtained by ANN (Eqs (3) and (4)) model for pine trees, and by statistical models STAT_1_ (Eqs (9) and (10)), STAT_2_ (Eqs (11) and (12)) are depicted in Figs 5–7. For the oak tree, the results are depicted in Figs 8–11.

STAT_1_ and STAT_2_ models showed the tendency for higher errors in the top (Fig 6) and bottom (Fig 5) parts of the stems notably for pine. Especially the STAT_1_ turned out to be worse in many cases, underestimating DBT at the top of the tree. For oak, the statistical models underestimated or overestimated the thickness of the bark in the middle part of the stem (Figs 8–11). There have been isolated cases where the neural network has estimated the thickness of the bark at the tree trunk slightly highest than the actual thickness (Fig 5). Closer to the top, STAT_1_ and STAT_2_ models smoothed the course of the DBT function for both pine and oak (Figs 7 and 8). ANNs matched the actual shape of the DBT function. Figs 6 and 10, show a better adaptation of the ANN model to atypical cases for both pine and oak. For oak (Figs 8–10) the tendency for STAT_1_ and STAT_2_ to smooth the shape is more pronounced than for pine. Also, atypical cases and outliers are projected worse (Figs 9–11). The models either underestimate the DBT thickness for the taller trees or overestimate them for, the lower trees (Figs 10 and 11).

## 4 Discussion

Tree growth is a complicated, nonlinear, and non-deterministic physiological process that also applies to tree bark. Usually, it is connected with the necessity to use unconventional methods for modeling. Our objective was to model the double bark thickness with two approaches used to predict the double bark thickness. The first method relates to the traditional and widely accepted nonlinear regression modeling. The second method uses the recursive ANN algorithm. The usefulness of this algorithm was tested due to its advantage over the traditional methodology. Nonlinear regression analysis estimates the coefficients in a mathematical equation that must be determined first. It requires a preliminary examination of the conditions of use. The role of each variable involved in modeling must be known. This distinguishes them from neural models. In a way, comparing the ANN and STAT_1,2_ models refers to testing the resistance of both methods to the input assumptions. Models that are more sensitive to atypical cases, stochastic disturbances, sample randomness disturbances, or measurement errors give the effect of larger systematic and forecast errors.

Modeling bark thickness requires capturing thickness differences due to environmental and within-tree variability. At work, we focused on individual differences. We chose two tree species that are representative both in terms of the importance of occurrence in Central Europe and differ in terms of DBT character as much as possible. We chose these two sets to see the differences in modeling. Pine grows in very different ecological conditions, which also affects the local variation in the thickness of the bark of these trees. On the other hand, oak has a very thick bark among deciduous trees with a very irregular course. In both cases, modeling with classical methods is difficult and requires removing outliers, dividing into age groups [24] or other treatments that facilitate obtaining precise models. However, the accuracy of model prediction does not always correspond to its universality, which is equally important from a practical point of view [25]. Choosing an appropriate modeling method should take into account both the trend and the dynamics of the phenomenon. In our research, we considered trees of different ages, including those with an irregular profile. We did not make any additional steps to remove outlier trees from the set. This way, we wanted to test the ability of the recursive model to adapt to the natural diversity and dynamics of the feature, which is the thickness of the bark. Primarily the oak is characterized by high irregularity in the stem morphology. Sometimes the bark of the oak in the upper parts of the stem is thicker than in the lower parts. According to the results of our analysis, the ANN model turned out to be better in terms of the ability to detect complex patterns and outliers. On individual trees, the differences in bark thickness depend on the position along the bole. When comparing tree taper over and under the bark, the functions are not parallel. The curvature of the outside-bark diameters function is greater than the inside-bark diameters function, so the ratio of bark decreases with height. On the other hand, above the relative height of 60 percent, the tapering of the stem under the bark is larger, so the ratio of bark in the tree’s thickness increases [5]. Especially in pine, the differences at the base of the tree are marked. Pine is characterized by a large thickness of the bark at the trunk. The rest of the tree is much thinner and has less variation. This results in a function mismatch at the bottom of the tree, where the bark is thickest. Incorrect bark estimation at this point is equivalent to the greatest inaccuracy of the estimation of the bark volume [26]. New research shows that a combination of stem taper function and bark thickness model (called the two-stage method) is suggested to predict DBT, especially in upper and lower portions of the tree stem. [27]. The ANN recursive model also estimated DBT much better than the non-linear function in the ranges up to 1 m high at the trunk and also 1–2 meters from the top of the tree. It would be very interesting to compare the two-stage method and ANN recursive model in future research. The stem taper equations that express mathematical relationships between tree height and diameter are essential for quantifying bark thickness as well as stem volume. A number of taper functions have been developed for different tree species with various forms including linear mixed functions, variable form taper functions, segmented polynomial taper functions, and also machine learning approaches including artificial neural network [28]. Based on these equations, bark thickness can then be calculated. Recursive MLP and RBF networks have been used to define the diameter prediction and volume calculations of Eucalyptus clones [24]. The results of Soares (2012) model were characterized by the accumulation of errors in the top part of the trees. The model was also developed for trees obtained from the clonal genetic material of the same age. In our work, the trees selected across stands varied in terms of age and site conditions, which was an additional complicating factor for the model. Deciduous trees are rarely modeled because of the difficulty associated with form diversity. The random forest and neural network models, used by Nunes and Görgens (2016) included 72 deciduous tree species. Input data were classified into three types and used as a qualitative variable. The information on the origin of a tree was crucial for the model because of the diversity of stem shapes in the different sites. Two multi-layer perceptrons were calibrated in the context of regression analyses and were found to be more accurate than the traditional taper equation, especially with regard to the prediction of diameter on lower and thicker portions of the stem or diameters of large trees. We also observed a good ANN model prediction for lower trees compared to the statistical models. In the works of Nunes and Görgens (2016) and also Nickolas (2016), prediction errors for smaller trees or diameters on the mid-range or upper portions of the stem were usually underestimated. In this regard, recursive models tend to be more representative of all data sets.

The results of our study show that it detects exceptional cases better than the standard models. Our findings are consistent with the recommendation of ANN models for describing the shape of a tree without bark. Recursive networks have not been used in DBT modeling so far. It seems that the ANN methodology can work very well for the unusual and challenging problems often associated with DBT prediction.

## 5 Conclusions

Under the conditions tested here, the recurrent ANNs are more suitable than regression models in the estimation of bark thickness. The best ANNs model for both pine and oak includes a Bayesian Regularization training algorithm with a single past value.

Time series is a suitable approach for capturing bark thickness variation for trees of diverse morphological types which are more common in deciduous species, like oak. ANN recursive model is more appropriate than regression models for estimating the bark thickness at the bottom and top of a tree, where classic models tend to overestimate or underestimate values, especially for pine.

Furthermore, the polynomial regression equation models either underestimate the bark thickness for the taller trees or overestimate them for, the lower trees. The ANN recursive model had an advantage in estimating such atypical trees. In particular, better estimates were for trees of less than average height. Such models are suitable for describing irregular cases so that they better describe the complex data set.

Additionally, ANN models can be improved by e.g. increasing the learning set, better fitting the model structure to the shape topology, changing the nonlinear transition function, or changing the learning parameters. The analyses performed, therefore, point to ANN as a universal tool for describing bark thickness. The presented ANN recursive models can be applied in the future to predict bark thickness in other species or regions.

## Supporting information

S1 Data (XLSX)

S2 Data (XLSX)
